# Supplementary material for: Signaling Pathways Impact on Induction of Corneal Epithelial-like Cells Derived from Human Wharton’s Jelly Mesenchymal Stem Cells
Source: Int J Mol Sci. 2022 Mar 12;23(6):3078. doi: 10.3390/ijms23063078 (PMC8949174; doi:10.3390/ijms23063078)
Supplement: Supplementary file 1 [file ijms-23-03078-s001.zip › ijms-1609458-supplementary.pdf]

International Journal of *Molecular Sciences*

MDPI

Supplementary Materials

Signaling pathways impact on induction of corneal epithelial-like cells derived from human Wharton's jelly mesenchymal

Contents

Figure S1. Effect of RA treatment on cell morphology.

Figure S2. PAX6 mRNA expression during CEC differentiation from WJ-MSCs.

Table S1. Antibodies used for immunofluorescent, flow cytometry and Western blot.

Table S2. Primers used for qPCR.

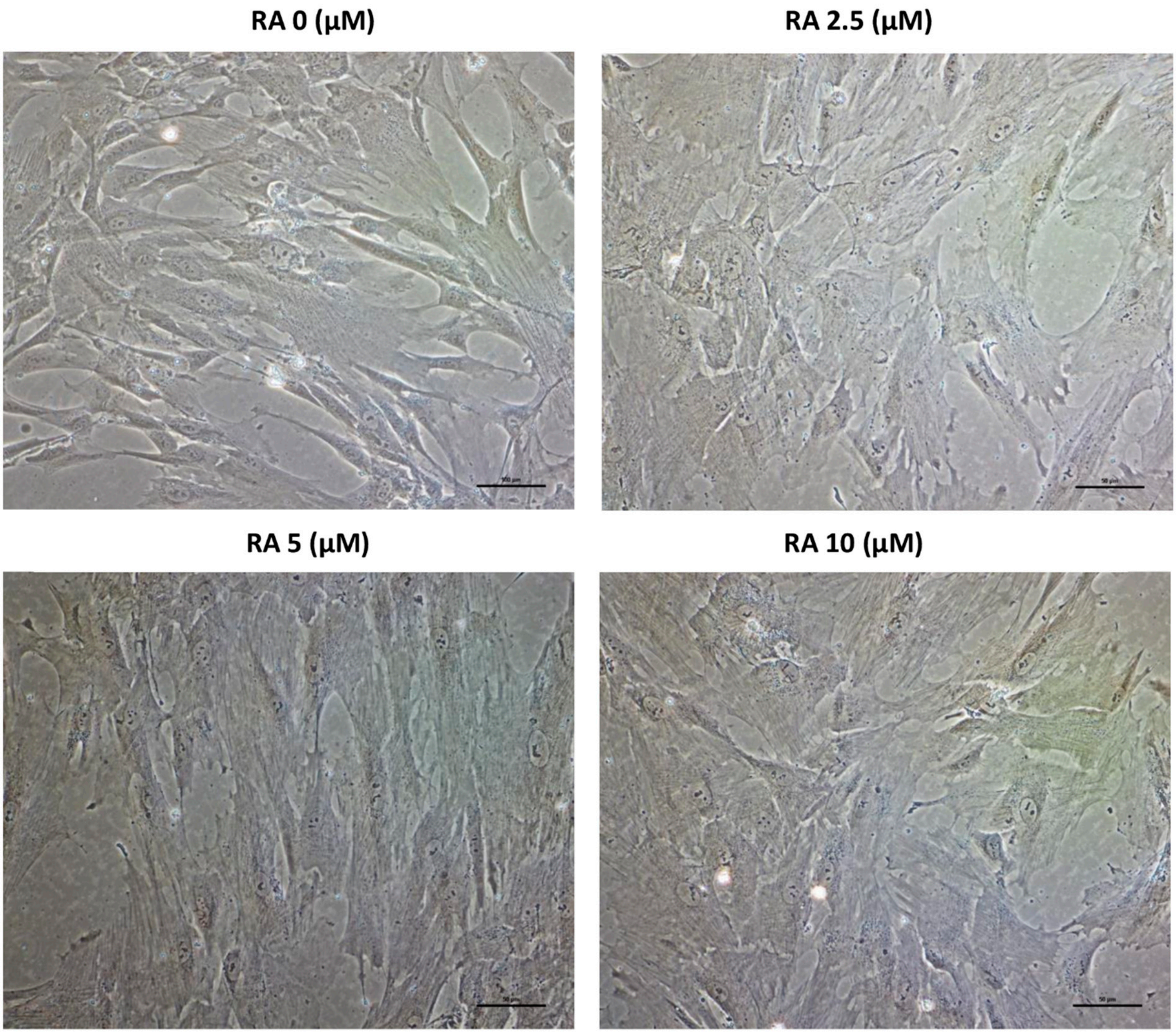

Figure S1. Effect of RA treatment on cell morphology. Scale bar, 50µm.

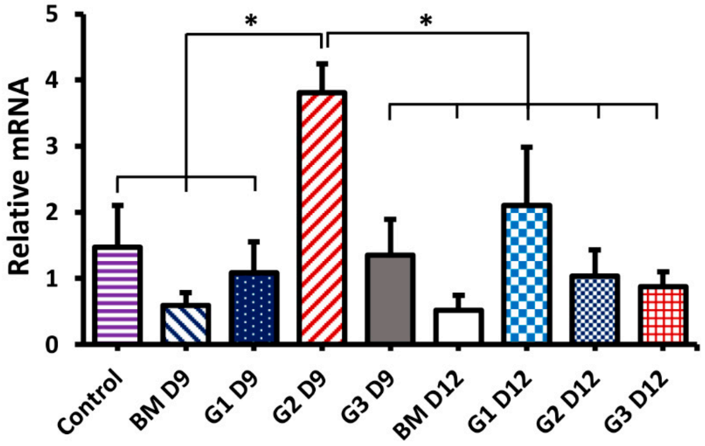

Figure S2. PAX6 mRNA expression during CEC differentiation from WJ-MSCs. Data are presented as mean + SEM. * $p < 0.05$.

Table S1. Antibodies used for immunofluorescent, flow cytometry and Western blot.

Antibodies	Companies	Cat #
PE mouse anti-CD105	BioLegend	323206
APC/Cy7 mouse anti-CD90	BioLegend	328132
APC mouse anti-CD73	BioLegend	344006
FITC mouse anti-CD45	BioLegend	368508
FITC mouse IgG, isotype Ctrl	BioLegend	400109
APC mouse IgG, isotype Ctrl	BioLegend	400120
PE mouse IgG, isotype Ctrl	BioLegend	400113
PE mouse anti-CD34	Beckman Coulter	A07776
Rabbit anti-ZO-1	Thermo Fisher Scientific	61-7300
Mouse anti-E-cadherin	Abcam	ab231303
Rabbit anti-β-catenin	Sigma-Aldrich	06-734/NA
Mouse anti-β-actin	Affinity Biosciences	T0022
Rabbit anti-Smad1/5/8/9	Abcam	ab13723
Rabbit anti-phospho-Smad1/5/8	Sigma-Aldrich	AB3848-I
Rabbit anti-Smad2/3	Sigma-Aldrich	07-408
Rabbit anti-phospho-Smad2/3	Sigma-Aldrich	SAB4504208
FITC mouse anti-Cytokeratin19	Thermo Fisher Scientific	MA5-28646
Mouse anti-Cytokeratin 12	Santa Cruz Biotechnology	sc-515882
Rabbit anti-ABCG2	Abcam	ab229193
Goat anti-mouse IgG HRP	Abcam	ab6789
Goat anti-rabbit IgG HRP	Abcam	ab6721
Goat anti-mouse IgG Alexa Flour 568	Thermo Fisher Scientific	A-11031
Goat anti-rabbit IgG FITC	Thermo Fisher Scientific	F2765

Table S2. Primers used for qPCR.

Gene	Primer sequence (5′ – 3′)	Product length

	Forward	Reverse	(bp)
hCTNNB1	CTGAGGACAAGCCACAAGATTACA	TGGGCACCAATATCAAGTCCAA	121
hABCG2	GTGCACATGCTTGGTGGTCTTGTT	ACCTCGGTCTTAACCAAAGGCTCA	159
hTP63	GCTCTGAAATCTTCCCATGCAT	ACATTACCTTTTAGAGCCACGC	106
hKRT3	CTCCAGATAAAGAGCACGCATC	CGGAGAGAAGAGCCTGAAATTC	203
hKRT12	TATTCTCTCGGGCAATGATGGA	TTGCTGTAATCGCTCTGTGAAG	201
hKRT15	GGAGGTGGAAGCCGAAGTAT	GAGAGGAGACCACCATCGCC	194
hKRT19	CTGCGGGACAAGATTCTTGGT	CCAGACGGGCATTGTCGAT	73
hPAX6	TCTTTGCTTGGGAAATCCG	CTGCCCGTTCAACATCCTTAG	167
hGAPDH	TGCACCACCACCTGCTTAGC	GGCATGGACTGTGGTCATGAG	87
